# Juvenile survival and movements of two threatened oceanic sharks in the North Atlantic Ocean inferred from tag‐recovery data

**DOI:** 10.1002/ece3.10198

**Published:** 2023-06-21

**Authors:** Gonzalo Mucientes, Albert Fernández‐Chacón, Nuno Queiroz, David W. Sims, David Villegas‐Ríos

**Affiliations:** ^1^ Instituto de Investigaciones Marinas, Consejo Superior de Investigaciones Científicas (IIM‐CSIC) Vigo Spain; ^2^ Centro de Investigação em Biodiversidade e Recursos Genéticos, InBIO Laboratório Associado (CIBIO‐InBIO) Universidade do Porto Vairão Portugal; ^3^ BIOPOLIS Program in Genomics, Biodiversity and Land Planning CIBIO Vairão Portugal; ^4^ Department of Natural Sciences, Centre for Coastal Research University of Agder Kristiansand Norway; ^5^ North Atlantic Marine Mammal Commission Tromsø Norway; ^6^ Marine Biological Association Plymouth UK; ^7^ Ocean and Earth Science, National Oceanography Centre Southampton University of Southampton Southampton UK

**Keywords:** blue shark, dispersal behavior, fishing mortality, North Atlantic, shortfin mako, survival, tag‐recovery data

## Abstract

Understanding population dynamics, movements, and fishing mortality is critical to establish effective shark conservation measures across international boundaries in the ocean. There are few survival and dispersal estimates of juveniles of oceanic shark species in the North Atlantic despite it being one of the most fished regions in the world. Here we provide estimates of dispersal, survival, and proportion of fishing mortality in the North Atlantic for two threatened oceanic sharks: the blue shark (*Prionace glauca*) and the shortfin mako shark (*Isurus oxyrinchus*). Our results are based on multi‐event models applied to tag‐recovery data of 700 blue sharks and 132 shortfin makos tagged over a decade. A total of 60 blue sharks (8.57% of tagged) and 30 makos (22.73%) were recovered by the longline fishery between 2009 and 2017. Tag‐reporting rate (percentage of returned information when a tagged shark was caught) was estimated to be high (0.794 ± 0.232 SE). Mean annual survival, as predicted from the models, was higher for blue shark (0.835 ± 0.040 SE) than for shortfin mako (0.618 ± 0.189 SE). Models predicted that fishing caused more than a half of total mortality in the study area for both species (0.576 ± 0.209), and more than a third of tagged individuals dispersed from the study area permanently (0.359 ± 0.073). Our findings, focused mainly on juveniles from oceanic areas, contribute to a better understanding of shark population dynamics in the North Atlantic and highlight the need for further conservation measures for both blue shark and shortfin mako, such as implementing efficient bycatch mitigation measures and static/dynamic time–area closures in the open ocean.

## Graphical abstract

Understanding population dynamics and fishing mortality is critical to establish shark conservation measures. We use tag–recapture data of >800 individuals and multi‐event models to investigate the fate of juvenile blue shark and shortfin mako. Fishing is the major source of mortality in both species.

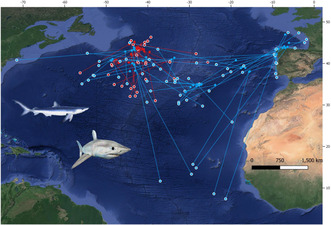

## 1 INTRODUCTION

Oceanic sharks are among the widest‐ranging animals in the ocean, typically moving across whole ocean basins and throughout a major part of the water column (0–2000 m; Queiroz et al., 2019). As for most elasmobranchs, the life‐history strategies of oceanic sharks are characterized by slow growth and late sexual maturity, which results in low fecundity and population productivity (Dulvy et al., 2021). Surviving through the long juvenile phase is therefore crucial to ensure the sustainability of populations (Kinney & Simpfendorfer, 2009). This is especially important for populations of oceanic sharks hampered by human activities such as fisheries, which can reduce reproductive opportunities for adults under scenarios of high fishing mortality (Camhi & Pikitch, 2008; Dulvy et al., 2021; Pacoureau et al., 2021).

Due to a lack of demographic and life‐history information, existing stock assessments of pelagic sharks are most commonly based on catches and/or catch‐at‐age data, which usually results in great uncertainty around the estimated parameters (Carvalho et al., 2018; Cortés & Brooks, 2018). Understanding the fate of sharks (e.g., their survival, mortality, and dispersal) is also required to accurately estimate population growth and total allowable catch for harvested oceanic sharks. In particular, determining the fate of the juvenile portion of the stocks of oceanic sharks with low fecundity is needed to understand which proportion of the population reaches the mature stock and can therefore contribute to the subsequent generation (Benson et al., 2018).

Traditionally, mark–recapture studies have been based on adults and coastal areas (Kohler & Turner, 2001). In this study, we used mark–recapture data to investigate the fate of two species of oceanic sharks in the North Atlantic with a main focus on juveniles. The blue shark (*Prionace glauca*) and the shortfin mako shark (*Isurus oxyrinchus*) are distributed throughout tropical and temperate waters from the surface to ~1800 m depth (Mucientes, 2023; Sims et al., 2018; Vedor et al., 2021). In the North Atlantic, both species are heavily fished (Campana et al., 2016; Queiroz et al., 2016, 2019; Sims et al., 2018), with catches of 36,500 tonnes and 3800 tonnes for blue and mako shark per year respectively (2007–2017; ICCAT, 2023), which has resulted in severe population declines in the last four decades (Dulvy et al., 2021; Pacoureau et al., 2021; Sims et al., 2021). Among the oceanic sharks, blue sharks have one of the highest population growth rates, with an age of maturity of 4–6 years and a litter size of 35–44 embryos (Dulvy et al., 2008). This life‐history strategy has likely contributed to a slower decline in the relative abundance of blue sharks in the North Atlantic over the past 50 years compared with other oceanic sharks, despite high fishing intensity (Pacoureau et al., 2021). Currently, there is a limitation in place, based on total allowable catches (TAC), for North and South Atlantic and, according to ICCAT (2020), the stock is “not overfished” and “overfishing is not occurring.” However, the species has been classified as “near threatened” globally by IUCN (Rigby, Barreto, Carlson, Fernando, Fordham, Francis, Herman, et al., 2019). In contrast, shortfin mako matures at a remarkably late age (7.5 years in males and 18–22 years in females (Natanson et al., 2006, 2020; Rosa et al., 2017; Yokoi et al., 2017)) and have a litter size of 8–12 embryos (Dulvy et al., 2008), which results in slow population growth. As a result, populations of shortfin mako have shown marked declines in abundance since 1970 that are attributed to overfishing (Pacoureau et al., 2021); indeed, ICCAT considers that the North Atlantic population is “overfished” with “overfishing still occurring” (ICCAT, 2019). Furthermore, shortfin mako is considered “Endangered” globally in the IUCN Red List assessment (Rigby, Barreto, Carlson, Fernando, Fordham, Francis, Jabado, et al., 2019).

Mark–recapture studies represent a valuable and cost‐effective means to obtain information about the life history and behavior of oceanic sharks (Kohler & Turner, 2001, 2019). Mark–recapture has been used to analyze the distribution of sizes and sex ratios in populations of coastal and oceanic sharks, such as Caribbean reef shark *Carcharhinus perezi* (Talwar et al., 2022) and great white shark *Carcharodon carcharias* (Kanive et al., 2021), to develop indices of relative abundance in zebra shark *Stegostoma fasciatum* (Dudgeon et al., 2008, 2013), to provide data on the population structure of whale shark *Rhincodon typus* (Rohner et al., 2022), and to inform international fisheries management organizations (Cortés & Brooks, 2018). Mark–recapture studies on blue shark and shortfin mako conducted in the Atlantic Ocean have been successful in collecting information on both short‐ and long‐term movements and migrations (Queiroz et al., 2005), growth rate, reproductive behavior, and for identifying mating and nursery areas (Kohler & Turner, 2019). Here, we expand the existing knowledge by specifically addressing movement behavior, to determine survival, dispersal, and mortality of juveniles of blue shark and shortfin mako in the Atlantic Ocean. Our results contribute to a more complete understanding of population growth and thus sustainability in these threatened species.

## 2 MATERIALS AND METHODS

### 2.1 Study area and tagging

Tagging of blue shark and shortfin mako was performed between 2007 and 2017 under the framework of the Cooperative Shark Tagging Program (CSTP, https://repository.library.noaa.gov/view/noaa/22731). The CSTP is a collaborative effort between recreational anglers, the commercial fishing industry, and scientific researchers to understand the movements and the life history of Atlantic shark species. It is managed by the Northeast Fisheries Science Centre, of the National Oceanic and Atmospheric Administration (NOAA).

**FIGURE 1:**
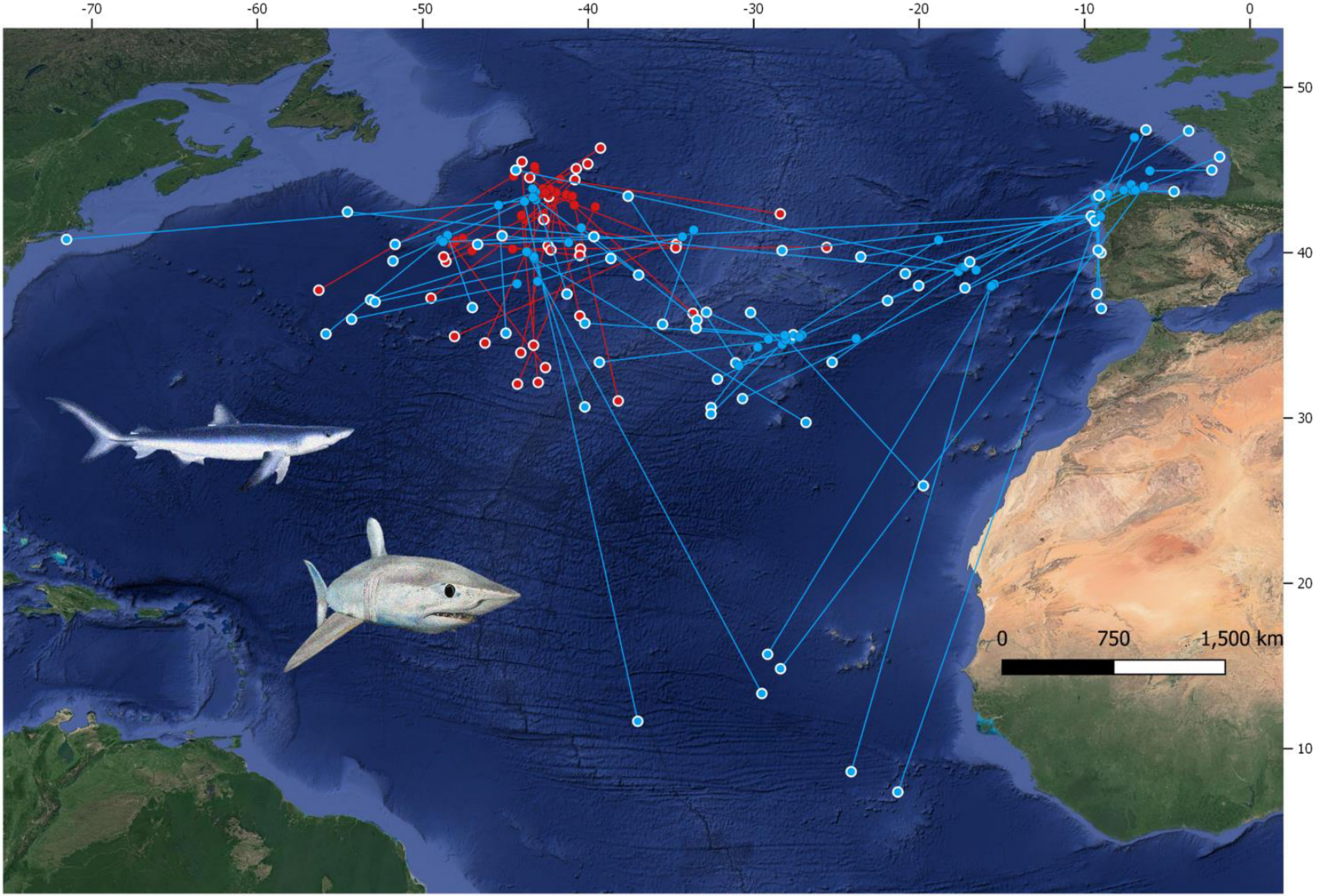
Capture and recovery locations (dots with external white line) of shortfin makos (red dots, bottom picture) and blue sharks (blue dots, upper picture). Yellow lines join the tagged and recovery locations.

Blue shark and shortfin mako were captured as target species (together with other species such as swordfish, *Xiphias gladius*). Both species were tagged by commercial fishers on board the Spanish longline fleet in the central North Atlantic (mainly west and south of the Azores islands); and mainly, by sport fishers (rod and reel) in coastal areas of Iberia (Figure 1). Fishers were trained in handling, tagging, and collecting data according to the procedures of the CSTP. The information recorded during tagging included species, size (fork length, FL), sex, date, gear type, and location of tagging. Based on size at maturity of blue (215 cm total length, TL; Dulvy et al., 2008) and shortfin mako, (200/280 cm TL male/female; Dulvy et al., 2008), most of the tagged individuals were likely juveniles at the time of capture. Conventional numbered dart tags (Kohler & Turner, 2001) were implanted in the dorsal musculature near the base of the first dorsal before sharks were released. This type of tag is highly visible to fishers and observers to increase the likelihood of sighting the tag upon the capture of the shark; furthermore, it has a small capsule at the posterior end containing detailed return instructions. Longline vessels and scientific observers reported the recoveries (Figure 1). Our study area thus corresponds to the area of the North Atlantic where both tagging and recoveries occurred.

### 2.2 Tag‐recovery, data analysis, and modeling approach

To estimate survival of the tagged sharks, the tag‐recovery data of blue shark and shortfin mako were used to construct two encounter history datasets (one for each species) that contained, for each year of the study period, information on whether the individual remained tagged or had been captured and the tag returned in that year. Since the data were collected opportunistically without a well‐defined annual sampling season, we adapted our recovery records to the classical encounter history format of discrete annual sampling occasions. The months of February through October were chosen as our annual sampling season because most tagging events occurred during that period of the year (100% of shortfin makos and 85% of blue sharks were initially captured during that period; Tables 2 and 3). Tag recoveries within a sampling season were assigned to the season's year, whereas recoveries taking place out of the sampling season were assigned to the next year (for a similar procedure see Fernández‐Chacón et al., 2015). Multi‐event modeling approach (Pradel, 2005), a type of hidden Markov model, was used to link tag recoveries to a series of underlying individual states defined in the model structure (see below and Appendix S1). This modeling approach has been successfully applied to mark‐recapture data of other marine species such as Atlantic cod (Fernández‐Chacón et al., 2015, 2016; Kleiven et al., 2016).

**FIGURE 2:**
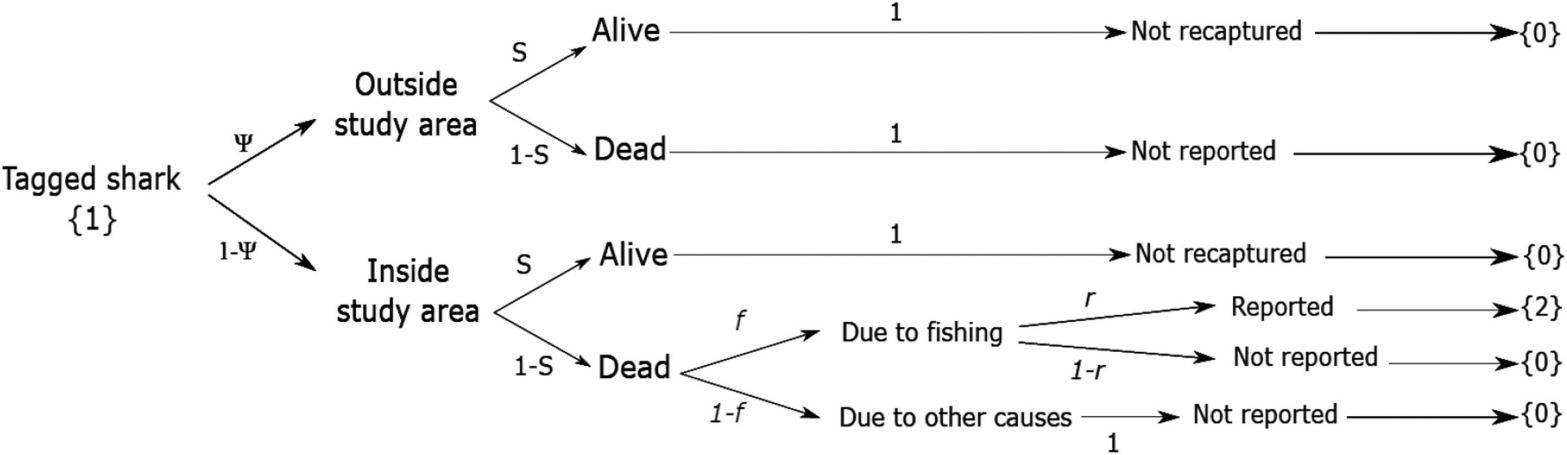
Diagram showing the model pattern used in the analysis of the encounter data. Each step represents a different model parameter and the whole sequence links both ecological (*ψ*, *S*, *f*) and observational processes (*r*) to the different events contained in the individual encounter histories (the numbers between brackets).

Our encounter data consisted of three types of observations or “events,” codified as follows: “not encountered” (0), “captured for the first time” (1), and “recovered dead” (2). From this set of events, we estimated annual individual survival, fishing mortality proportions, dispersal probabilities, and tag‐reporting rates. We did so by constructing a model pattern based on transition matrices that linked the observed events to transitions between possible underlying states, in which individuals may be found at a given occasion (Figure 2). In this model individuals could transition among six states: alive in the study area (“I”), alive outside the study area (“O”), dead by fishing in the study area (“DFI”), dead by other (unknown) causes in the study area (“DUI”), dead outside the study area (“DUO”), and dead for a long time (“†”). By “inside the study area” we mean the area of the ocean where sampling occurred, whereas “Alive outside the study area” is a mathematical concept, rather than a geographical area, that allowed us to account for the possibility of some tagged individuals moving into a state where they remain alive but unobservable. Note that states “O”, “DUI”, and “DUO” are not observable and can only be linked to the event “not encountered” (see below and Figure 2): here, DUI and DUO states reflect unobservable but recently dead individuals, whereas state “O” indicates that the individual is alive but unavailable for sampling. The state “†” is an additional unobservable dead state that was also included in the model definition to distinguish the observed recoveries or “newly dead” individuals from the unobservable “long‐time dead” ones (see Lebreton et al., 1999). This classification allows estimating mortality proportions due to fishing and tag‐reporting rates (see below). Between each sampling occasion, sharks can change state according to the transitions shown in Figure 2. The probabilities associated with each change of state are defined in the full transition matrix (Φ), which can be written as:

$$\Phi =\left(\begin{array}{ccccccc} \text{From}/\text{to} & I & O & \mathrm{DFI} & \mathrm{DUI} & \mathrm{DUO} & †\\ I & S\left(1-\psi \right) & \psi & f\left(1-S\right) & \left(1-f\right)\left(1-S\right) & 0 & 0\\ O & \psi & S\left(1-\psi \right) & 0 & 0 & 1-S & 0\\ \mathrm{DFI} & 0 & 0 & 0 & 0 & 0 & 1\\ \mathrm{DUI} & 0 & 0 & 0 & 0 & 0 & 1\\ \mathrm{DUO} & 0 & 0 & 0 & 0 & 0 & 1\\ † & 0 & 0 & 0 & 0 & 0 & 1 \end{array}\right)$$

where, *S*: the annual survival probability; *Ψ*: the probability of moving from one area to another. Two types of movement transitions are possible:

*Ψ*_*I→O*_: emigration (from inside to outside the study area, that is, areas where vessels that participated in the study traveled making tag recoveries still feasible).

*Ψ*_*O→I*_: immigration (from outside to inside the study area).

*f*: the probability of death due to fishing given that an animal has died in the study area.

These model parameters could be estimated separately by splitting the full transition matrix into a three‐step series of transition matrices representing dispersal, survival, and cause‐specific mortality processes, respectively (see Appendix S1). Our model pattern assumes that ecological processes occur before the observational ones, with dispersal being the first step in our sequence of transition matrices and survival the second. If an individual dies in the study area, it can transit to two dead states (one observable and one unobservable, see Figure 2), thus estimating the proportion of deaths due to fishing separately from other causes of mortality. Finally, the third and last step corresponds to the observational process and allows us to estimate event probabilities. Matrix *E* shows the event probabilities that link the biological states (rows) with the observations (columns).

$$E=\left(\begin{array}{cccc} \text{From}/\text{to} & 0 & 1 & 2\\ I & 1-p & p & 0\\ O & 1 & 0 & 0\\ \mathrm{DFI} & 1-r & 0 & r\\ \mathrm{DUI} & 1 & 0 & 0\\ \mathrm{DUO} & 1 & 0 & 0\\ † & 1 & 0 & 0 \end{array}\right)$$

where, *p*: the recapture probability of a marked animal that is alive; *r*: the reporting probability of a marked animal dead by fishing.

Events “1” and “2” are directly linked to model states “I” and “DFI” (i.e., they can only happen in these states) but event “0” (not encountered) arises from imperfect detection (see also Figure 2) and can be related to any possible underlying state in our probabilistic model. Because non‐fishing deaths and those occurring outside the study area were never reported, their corresponding states can only be linked to event “0” (see also Figure 2). Given that no animals were recaptured alive in our study (only dead sharks were reported), the recapture probability (*p*) was always fixed to zero in our modeling.

### 2.3 Goodness‐of‐fit test and model construction

Multi‐event models were built and fitted to the data using the program E‐SURGE (Choquet & Nogue, 2010). Prior to the model selection process, a Goodness‐of‐fit test was conducted to check if our data met the assumptions of a departure model that considers all parameters to be state and time‐dependent, namely the Arnason–Schwarz model (Pradel et al., 2003). Goodness‐of‐fit tests were performed using U‐CARE (Choquet et al., 2009), a statistical program that by means of contingency tables helps users to detect sources of lack of fit in their encounter data, which are mainly caused by differences in survival and detection probabilities among individuals. To correct for those sources of lack of fit, we calculated an overdispersion coefficient or *ĉ* (the sum of chi‐square results for each test divided by the total number of degrees of freedom) that was applied to the analyses in E‐SURGE.

Model selection was based on Akaike's information criterion corrected for overdispersion (Quasi‐AIC or QAIC), and we retained as good candidate models those showing the lowest QAIC values (Beier et al., 2001). Models differing in <2 points of QAIC from the top‐ranked one (ΔQAIC <2) were also considered good candidate models (i.e., statistically equivalent).

Encounter data from both species were analyzed together under the same multi‐event modeling approach. By analyzing both species together, we increased the amount of data available for making statistical inference allowing us to build models with more mathematical parameters, testing biological hypotheses, and quantifying rates that would not have been tested nor quantified otherwise. The model selection process departed from a general model considering full time (year) and group (species) effects on annual survival (*S*), fishing mortality proportions (*f*), and tag‐reporting rates (*r*) of dead sharks. State effects were not tested in S and survival was assumed to be identical between the two “alive” states (SI = SO). Time and group effects were not tested on dispersal transitions (*Ψ*_*I→O*_, *Ψ*_*O→I*_); instead, these parameters were modeled following four hypotheses regarding shark movement in/out of the study area: (i) No movement (*Ψ*_*I→O*_ *= Ψ*_*O→I*_ *= 0*), (ii) Markovian movement (*Ψ*_*I→O*_ *≠ Ψ*_*O→I*_), (iii) Random movement (*Ψ*_*I→O*_ = *Ψ*_*O→I*_), and (iv) Permanent emigration (*Ψ*_*I→O*_ *≠* 0, *Ψ*_*O→I*_ *= 0*). The testing of group (species) and time (year) effects focused on *S*, *f*, and *r* parameters. Modeling of *r* consisted of removing group and time interactions (“*”) and on testing constancy (“.”), time‐only (*t*), group‐only (species), and additive (+) time and group effects on this parameter until the most parsimonious (i.e., lowest QAIC) model structure was determined. Once a best structure for *r* was found, we kept that structure and repeated the same modeling process with *f* and *S* parameters until a consensus model, with the best supported structure for *S*, *f*, and *r*, had been retained. In both our departure model and in the subsequent modeling of *S*, *f*, and *r* parameters, we always kept immigration transitions fixed to zero (i.e., a permanent emigration structure). Alternative hypotheses regarding *Ψ* were also tested on the consensus model, to check whether they improved, or not, the retained model structure.

## 3 RESULTS

**FIGURE 3:**
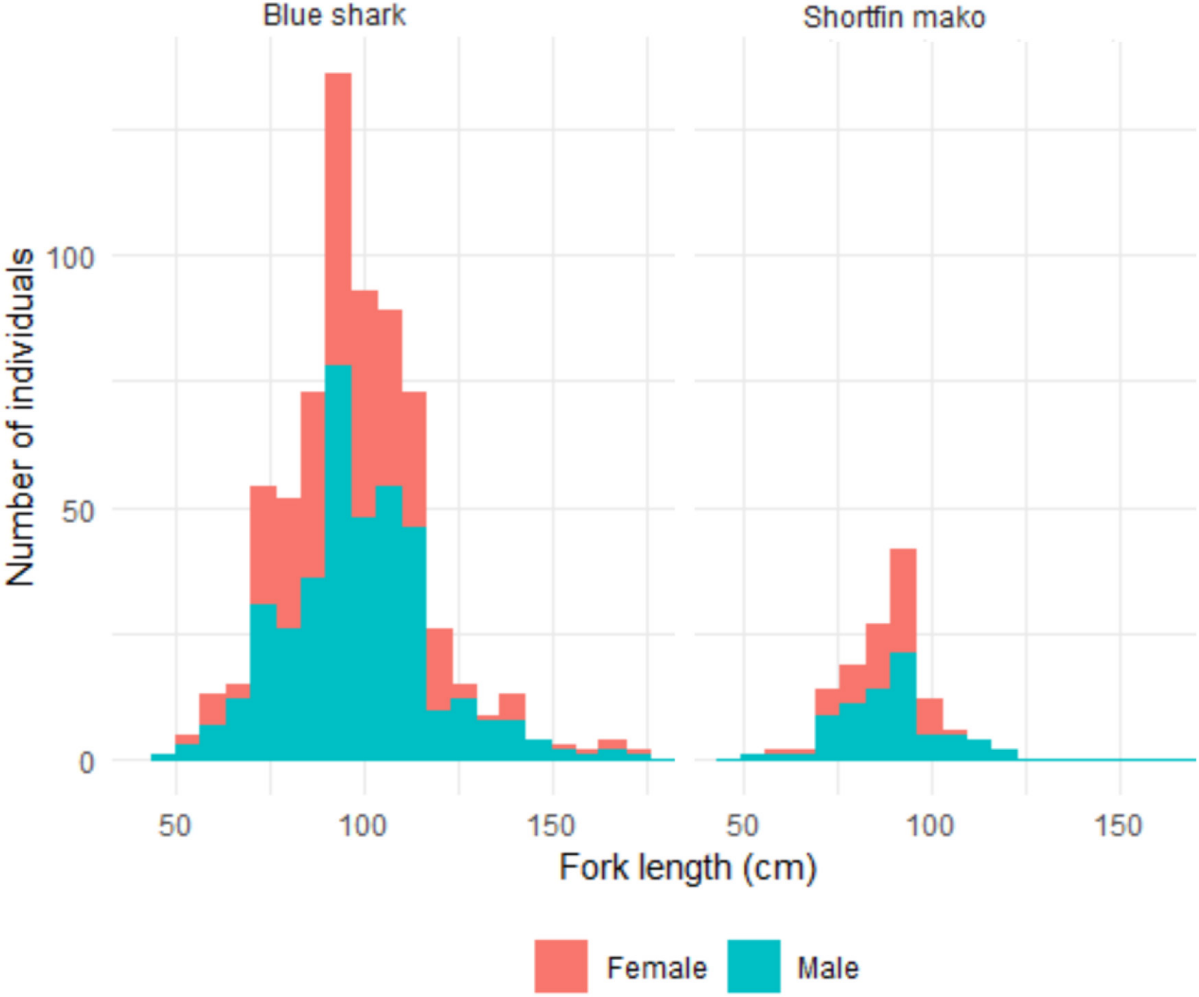
Length‐frequency distributions of shortfin mako (*Isurus oxyrinchus*) and blue shark (*Prionace glauca*) tagged in this study.

A total of 700 blue sharks and 132 shortfin makos were tagged (Table 1) of which 60 blue sharks (8.57% of tagged) and 30 shortfin makos (22.73%) were recovered between 2009 and 2017. The fork length of blue sharks ranged between 48 and 240 cm FL (mean = 97.17 ± 19.86 cm SD) and between 55 and 180 cm FL (mean = 87.98 ± 11.45 cm) in the case of shortfin makos (Figure 3). Time at liberty ranged between 42 and 2180 days for blue shark (total days = 31,970; days/shark = 45.67), Table 2; and 32 and 1118 days for shortfin mako (total days = 12,137; days/shark = 91.95), Table 3. The minimum distance traveled based on tagging and tag‐recovery positions ranged between 32.5 and 4046.4 km (1216.1 km ± 1003.5 km SD; total = 72,968 km, 104.24 km/shark) for blue shark (Table 2). In the case of shortfin mako, the minimum distance traveled ranged between 27.6 and 1607.7 km (784.2 km ± 407.6 km; total = 23,525.3 km, 178.22 km/shark; Table 3).

**TABLE 1:** Summary of capture and recovery data for shortfin mako and blue shark.

Species	Number of individuals tagged	Males/females	Sex unknown	Number (and %) of recoveries
*Shortfin mako*	132	75/58	1	30 (22.73%)
*Blue shark*	700	392/292	16	60 (8.57%)

**TABLE 2:** Tagging and recovery information of blue shark (*Prionace glauca*) obtained during this study. Diff FL= difference in fork length between tagging and recovery; Sex (female, 0; male, 1).

Tag ID	Latitude	Longitude	Date	FL (cm)	Sex	Days at liberty	Distance (km)	Diff FL (cm)
307,266	38.87	−17.62	26/11/2007	65	1			
307,266	42.47	−54.55	05/12/2009	165	1	740	3115.78	100
307,272	39.13	−17.35	26/11/2007	70	1			
307,272	45.00	−44.37	20/08/2008	115	1	268	2311.63	45
323,434	34.72	−27.72	23/02/2008	92	1			
323,434	38.00	−20.02			1		779.57	
323,435	34.93	−28.10	25/02/2008	95	1			
323,435	35.73	−40.20	12/02/2009	155	1	353	1100.52	60
323,444	35.02	−27.07	24/02/2008	100	1			
323,444	32.35	−32.18	15/09/2009	170	1	569	558.50	70
323,448	34.80	−23.80	21/02/2008	98	1			
323,448	33.33	−31.08	03/02/2010	200	1	713	690.04	102
323,461	34.45	−28.27	27/02/2008	86	1			
323,461	36.38	−30.18	07/12/2009	163	1	649	275.88	77
323,464	34.77	−29.10	26/02/2008	103	1			
323,464	36.40	−32.85	13/01/2009	127	1	322	384.65	24
323,470	33.15	−30.88	04/03/2008	110	1			
323,470	43.42	−37.58	25/07/2009	183	1	508	1281.59	73
323,474	34.28	−29.75	01/03/2008	103	1			
323,474	30.63	−32.57	15/01/2010	200	1	685	484.74	97
323,477	34.90	−28.12	28/02/2008	95	1			
323,477	25.90	−19.73	14/09/2009	160	1	564	1282.56	65
328,431	40.77	−18.82	12/12/2008	106	1			
328,431	35.03	−27.60	10/02/2009	112	1	60	999.26	6
328,436	39.15	−17.23	16/12/2008	95	1			
328,436	33.37	−25.25	05/03/2010	140	1	444	963.47	45
328,444	38.95	−16.55	17/12/2008	105	0			
328,444	47.37	−3.70	18/06/2010	170	0	548	1397.73	65
329,817	38.10	−15.45	05/02/2009	77	1			
329,817	40.00	−9.00	04/05/2009	91	1	88	595.52	14
330,013	34.73	−28.05	15/03/2009	100	1			
330,013	30.25	−32.57	10/01/2010	150	1	301	654.23	50
330,287	39.63	−43.25	23/04/2009	110	1			
330,287	29.73	−26.83	16/01/2010	150	1	268	1856.93	40
333,095	41.50	−40.42	22/06/2009	100	1			
333,095	35.93	−33.42	05/11/2009	130	1	136	866.60	30
333,337	43.20	−43.17	27/06/2009	108	1			
333,337	40.80	−71.53	31/08/2010	130	1	430	2347.55	22
333,348	43.67	−43.18	27/06/2009	114	1			
333,348	40.50	−51.67	16/04/2010	160	1	293	783.37	46
335,444	42.12	−9.20	11/07/2010	115	0			
335,444	39.45	−16.93	13/01/2011	140	0	186	714.97	
339,105	42.12	−9.20	11/07/2010	100	0			
339,105	40.15	−9.17	06/11/2010	101	0	118	219.07	1
330,005	34.88	−27.23	13/03/2009	135	1			
330,005	33.37	−39.32	21/03/2011	212	1	738	1124.48	77
339,922	42.22	−9.35	22/08/2010	90	1			
339,922	40.13	−28.28	24/04/2011	112	1	245	1597.94	22
330,098	40.00	−43.62	26/04/2009	96	1			
330,098	37.15	−53.15	27/04/2009	165	1	731	886.69	69
339,914	40.77	−48.92	07/07/2010	104	1			
339,914	35.08	−55.85	06/05/2011	118	1	303	876.54	14
323,479	35.00	−28.12	28/02/2008	110	0			
323,479	35.43	−33.48	13/03/2011	205	0	1109	489.51	95
335,434	42.17	−9.23	20/09/2009	150	1			
335,434	7.37	−21.28	27/11/2010	175	1	433	4046.41	25
333,341	43.35	−43.27	27/06/2009	103	1			
333,341	39.50	−51.80	12/05/2011	190	1	684	829.71	87
338,846	40.62	−41.18	25/04/2010	91	1			
338,846	41.02	−45.20	17/05/2011	131	1	387	341.16	40
339,911	41.05	−48.50	06/07/2010	119	0			
339,911	36.68	−47.00	01/04/2011	120	0	269	502.95	1
333,325	38.27	−43.05	04/10/2009	80	1			
333,325	39.65	−38.63	01/07/2011	115	1	635	411.91	35
336,626	40.97	−34.30	02/10/2009	115	1			
336,626	37.02	−52.88	29/05/2011	200	1	604	1661.01	85
339,900	40.67	−48.77	30/06/2010	106	1			
339,900	40.97	−39.65	07/07/2011	145	1	372	767.81	39
333,104	43.87	−43.35	27/06/2009	111	1			
333,104	37.50	−41.28	08/09/2011	200	1	803	729.07	89
339,127	38.12	−44.30	07/05/2010	96	0			
339,127	35.97	−54.30	21/03/2011	120	0	259	918.62	24
330,291	39.75	−43.28	23/04/2009	98	0			
330,291	30.67	−40.20	15/09/2011		0	875	1047.59	
329,827	37.93	−15.67	05/02/2009	85	1			
329,827	8.60	−24.10	11/01/2011		1	795	3369.76	
341,472	42.28	−9.30	12/06/2011	120	1			
341,472	39.75	−23.52	20/01/2012	120	1	222	1224.19	0
330,088	40.05	−43.72	25/04/2009	120	0			
330,088	13.33	−29.50	01/02/2012	190	0	1012	3279.65	70
338,827	43.80	−7.00	26/06/2011	112	1			
338,827	38.73	−20.83	15/03/2011	120	1	263	1283.86	8
345,201	44.95	−6.05	29/07/2011	100	1			
345,201	45.00	−2.30		110	1		295.00	10
338,826	43.77	−7.10	26/06/2011	104	0			
338,826	45.80	−1.83	16/06/2012		0	356	473.08	
336,620	41.37	−33.62	30/09/2009	86	0			
336,620	35.67	−35.50	16/04/2012	200	0	929	654.24	114
333,114	43.4	−43.3	28/06/2009	104	0			
333,114	38.7	−37	25/05/2012	164	0	1062	747.95	60
345,198	43.4	−9.38	26/07/2011	110	1			
345,198	36.6	−8.98	24/09/2012	150	1	426	751.34	40
341,471	42.3	−9.3	12/06/2011	82	1			
341,471	40.5	−46.7	12/11/2012	150	1	519	3099.49	68
350,590	47	−6.98	14/06/2012	86	0			
350,590	42.2	−9.58	07/09/2012	90	0	85	566.79	4
333,334	43.1	−43.9	26/06/2009	98	0			
333,334	11.7	−37	15/09/2012	190	0	1177	3558.92	92
348,728	42.3	−9.08	21/05/2012	92	1			
348,728	37.5	−9.25	20/05/2013	137	1	364	531.71	45
338,824	44.1	−7.18	27/06/2011	108	1			
338,824	37.1	−21.9	01/05/2013	140	1	674	1464.08	32
350,582	43.8	−6.92	01/08/2012	92	1			
350,582	37.9	−17.2		136	1		1086.25	44
351,160	43.55	−8.60	05/08/2012	112	0			
351,160	43.47	−9.12	17/08/2013	133	0	377	42.87	21
339,896	42.87	−45.42	27/06/2010	100	0			
339,896	35.13	−44.97	09/12/2014	185	0	1626	861.52	85
345,204	44.00	−6.40	29/07/2011	115	0			
345,204	14.83	−28.37	19/12/2014	190	0	1239	3852.47	75
329,823	37.98	−15.62	05/02/2009	90	0			
329,823	15.70	−29.15	25/01/2015	240	0	2180	2810.71	150
350,578	41.53	−9.38	24/07/2012	97	1			
350,578	31.17	−30.67	28/01/2015	180	1	918	2218.47	83
351,173	43.78	−7.63	02/08/2012	95	0			
351,173	43.70	−4.58	29/05/2014	128	0	665	245.17	33
339,111	42.15	−9.12	04/07/2015	240	1			
339,111	41.93	−9.38	15/08/2015	190	1	42	32.55	−50
371,859	42.18	−9.02	08/10/2016	160	0			
371,859	47.43	−6.28	22/09/2017	155	0	349	622.40	−5

**TABLE 3:** Tagging and recovery information of shortfin mako (*Isurus oxyrinchus*) obtained during this study. Diff FL=difference in fork length between tagging and recovery; Sex (female, 0; male, 1).

Tag ID	Latitude	Longitude	Date	FL (cm)	Sex	Days at liberty	Distance (km)	Diff FL (cm)
324,085	43.28	−42.07	03/07/2008	80	1			
324,085	36.17	−40.50	01/03/2010	140	1	606	802.19	60
324,086	43.33	−42.25	03/07/2008	90	1			
324,086	34.53	−46.23	16/01/2009	114	1	197	1037.06	24
324,090	43.62	−43.08	08/07/2008	80	1			
324,090	45.08	−40.72	05/08/2009	130	1	393	249.00	50
324,099	43.63	−42.50	11/07/2008	103	0			
324,099	43.40	−42.38	28/06/2009	170	0	352	27.60	67
324,101	43.68	−42.50	11/07/2008	95	1			
324,101	40.42	−42.42	22/10/2008	100	1	103	363.30	5
324,104	43.53	−42.87	11/07/2008	88	0			
324,104	40.22	−40.47	20/10/2009	150	1	466	418.97	62
324,105	43.70	−41.95	12/07/2008	83	0			
324,105	40.17	−42.25	09/09/2009	145	0	424	393.67	62
324,110	43.63	−41.93	14/07/2008	93	0			
324,110	33.95	−44.07	01/02/2009	120	0	202	1092.39	27
324,113	44.45	−41.68	17/07/2008	96	1			
324,113	42.35	−28.38	05/10/2009	135	1	445	1098.27	39
324,118	43.52	−41.32	18/07/2008	94	0			
324,118	37.25	−49.50	25/04/2009	120	0	281	981.90	26
330,084	40.12	−47.02	10/05/2009	80	1			
330,084	39.47	−48.60	11/06/2009		1	32	153.37	
333,121	42.32	−42.80	04/07/2009	60	1			75
333,121	44.42	−40.80	30/08/2009	75	1	57	284.00	
335,408	42.88	−40.83	31/08/2009	102	1			
335,408	31.03	−38.18	19/01/2010	120	1	141	1338.75	18
335,415	45.00	−43.22	06/09/2009	90	0			
335,415	36.33	−33.67	25/11/2009	110	0	80	1254.15	20
324,095	43.87	−42.73	09/07/2008	80	1			
324,095	44.55	−43.53	24/07/2009	130	1	380	99.02	50
330,094	40.15	−43.72	26/04/2009	120	1			
330,094	45.50	−44.00	28/07/2009	130	1	93	595.34	10
330,083	40.50	−48.22	06/05/2009	73	0			
330,083	33.05	−42.58	02/03/2010	100	0	270	968.13	27
324,109	43.85	−42.30	13/07/2008	91	0			
324,109	42.00	−42.68	02/02/2010	140	0	569	208.02	49
330,261	40.23	−44.57	24/04/2009	107	1			
330,261	40.32	−25.58	07/11/2009	135	1	197	1607.65	28
336,593	43.42	−40.95	13/09/2009	118	1			
336,593	39.75	−48.73	03/05/2010	117	1	232	764.32	−1
335,424	45.22	−43.23	10/09/2009	93	0			
335,424	34.40	−43.30	30/03/2010	120	0	201	1202.77	27
324,097	43.65	−42.60	10/07/2008	95	1			
324,097	40.50	−34.67	25/10/2009	146	1	472	741.89	51
335,405	42.78	−39.57	29/08/2009	90	0			
335,405	32.05	−44.27	24/02/2011	145	0	544	1263.14	55
335,418	44.63	−44.50	07/09/2009	103	1			
335,418	37.73	−56.28	30/05/2011	160	1	630	1247.30	57
330,082	40.70	−47.98	05/05/2009	90	1			
330,082	39.83	−40.50	01/11/2011	160	1	910	642.07	70
336,607	40.97	−34.35	25/09/2009	100	1			
336,607	34.93	−48.08	22/04/2012	140	1	940	1376.06	40
333,311	40.87	−47.58	14/07/2009	90	1			
333,311	46.33	−39.25	05/08/2012	180	1	1118	904.28	90
351,184	42.00	−43.82	15/06/2014	75	1			
351,184	45.37	−40.02	13/07/2015	125	1	393	483.39	50
351,177	42.18	−44.03	15/06/2014	79	0			
351,177	32.15	−43.02	24/01/2016	150	0	588	1118.85	71
351,179	42.27	−44.02	16/06/2014	99	0			
351,179	40.30	−34.70	14/09/2016	165	0	821	808.48	66

### 3.1 Goodness‐of‐fit testing and model selection results

The multistate Goodness‐of‐fit tests performed for the two‐species encounter history dataset yielded significant results and the *ĉ* coefficients resulting from each subset of data were all >1 (see Appendix S1). Such results indicated that the departure model used in the test (Arnason–Schwarz model) did not fit our data adequately and that overdispersion was present, yielding a global *ĉ* value of 1.89 that was applied as a correction factor when running the multi‐event models in E‐SURGE. In the multi‐event modeling we departed from a more complex model (model 16, Table 4; all model structures in this table) that considered different causes of mortality and accounted for dispersal outside the study area, so incorporating many potential sources of lack of fit. The highest ranked model structure (Model 1) was the one considering a permanent emigration movement strategy (Ψ_*I*→*O*_ ≠ 0, Ψ_*O*→*I*_ = 0), constant but different annual survival probability (*S*) between species, and constant and identical fishing mortality proportions (*f*) and tag‐reporting rates (*r*) between species. The parsimony of the initial departure model (Model 16) increased when both time and species effects were removed from r and f (Model 16 vs. Model 12 and Model 12 vs. Model 8). Removing time effects from S improved model structure (Model 8 vs. Model 1), but the removal of species effects did not (Model 5 vs. Model 1). Testing alternative structures on ψ (i.e., Markovian, Random or No movement) did not increase model parsimony either (Models 3, 4, and 6 vs. Model 1). A model with additive species and time effects on S was the second best‐ranked model of the set (Model 2), however, that model was 3.74 points of QAIC higher than the first‐ranked one (ΔQAIC >2) and thus not better supported nor statistically equivalent.

**TABLE 4:** Model selection results obtained in the multi‐event analysis of shark tag‐recovery data showing all model structures tested on dispersal transitions (*ψ*), survival (*S*), mortality proportions (*f*), and reporting rates (*p*).

Model	*ψ*	*S*	*f*	*r*	Np	QAIC	ΔQAIC
**1**	***Ψ***_***I→O***_ ***≠* 0, *Ψ***_***O→I***_ ***= 0***	**species**	**(.)**	**(.)**	**5**	**336.8337**	**0**
2	*Ψ*_*I→O*_ *≠* 0, *Ψ*_*O→I*_ *= 0*	species + t	(.)	(.)	13	340.5756	3.7419
3	*Ψ*_*I→O*_ *= Ψ*_*O*→*I*_ *= 0*	species	(.)	(.)	4	341.5431	4.7094
4	*Ψ*_*I*→*O*_ = *Ψ*_*O*→*I*_	species	(.)	(.)	5	342.2531	5.4194
5	*Ψ*_*I*→*O*_ *≠* 0, *Ψ*_*O*→*I*_ *= 0*	(.)	(.)	(.)	4	342.7725	5.9388
6	*Ψ*_*I*→*O*_ *≠ Ψ*_*O*→*I*_	species	(.)	(.)	6	344.1941	7.3604
7	*Ψ*_*I*→*O*_ *≠* 0, *Ψ*_*O*→*I*_ *= 0*	t	(.)	(.)	12	344.7159	7.8822
8	*Ψ*_*I*→*O*_ *≠* 0, *Ψ*_*O*→*I*_ *= 0*	species * t	(.)	(.)	23	355.2102	18.3765
9	*Ψ*_*I*→*O*_ *≠* 0, *Ψ*_*O*→*I*_ *= 0*	species * t	species	(.)	24	357.2102	20.3765
10	*Ψ*_*I*→*O*_ *≠* 0, *Ψ*_*O*→*I*_ *= 0*	species * t	t	(.)	32	367.5518	30.7181
11	*Ψ*_*I*→*O*_ *≠* 0, *Ψ*_*O*→*I*_ *= 0*	species * t	species + t	(.)	33	370.0281	33.1944
12	*Ψ*_*I*→*O*_ *≠* 0, *Ψ*_*O*→*I*_ *= 0*	species * t	species * t	(.)	42	385.0239	48.1902
13	*Ψ*_*I*→*O*_ *≠* 0, *Ψ*_*O*→*I*_ *= 0*	species * t	species * t	species	43	387.0266	50.1929
14	*Ψ*_*I*→*O*_ *≠* 0, *Ψ*_*O*→*I*_ *= 0*	species * t	species * t	t	51	403.0238	66.1901
15	*Ψ*_*I*→*O*_ *≠* 0, *Ψ*_*O*→*I*_ *= 0*	species * t	species * t	species + t	52	405.0238	68.1901
16	*Ψ*_*I*→*O*_ *≠* 0, *Ψ*_*O*→*I*_ *= 0*	species * t	species * t	species * t	61	423.0238	86.1901

*Note*: The number of mathematical parameters (Np), Quasi‐Akaike information criterion values (QAIC), and difference in QAIC between a given model and the top‐ranked model (ΔQAIC) are also given. The best model of the set is shown in bold.

### 3.2 Survival, emigration, fishing mortality, and reporting rate.

Annual survival values (as a proportion of 1 that equates to total survival) were obtained from the optimal model and differed between species, being higher for blue shark (*S*_blue_ = 0.835 ± 0.040 SE, [0.757–0.913] 95% CI) than for shortfin mako individuals (*S*_mako_ = 0.618 ± 0.189, [0.248–0.988]). All other parameters were identical between species: the proportion of mortality that can be attributed to fishing (*f*) was 0.576 ± 0.209 (95% CI: 0.166–0.986), tag‐reporting rate was 0.794 ± 0.232 (0.339–1.000), and permanent emigration (Ψ_*I*→*O*_) was 0.359 ± 0.073 (0.216–0.502; as a proportion of 1 that equates to total emigration).

## 4 DISCUSSION

By using an extensive tag‐recovery dataset of more than 800 individuals, mainly juveniles, we were able to estimate important demographic parameters of two heavily exploited oceanic sharks: blue shark and shortfin mako shark. Survival rate was moderate for shortfin mako and high for blue shark; fishing mortality represented the major source of mortality for both species and one third of the individuals dispersed from the North Atlantic permanently. Taken together, these results emphasize the high impact of fishing on the juvenile portion of the stock of both species and the need for conservation and management measures for these two oceanic sharks.

The annual survival rate of the juvenile fraction of blue sharks and shortfin mako estimated in this study (*S*_blue_ = 0.835 ± 0.040; *S*_mako_ = 0.618 ± 0.189) falls within or close to the range of previously reported values for the species in the North Atlantic [blue shark, 0.530–0.910 (Aires‐Da‐Silva & Gallucci, 2007); shortfin mako, 0.705–0.873 (Wood et al., 2007)]. The age and size at 50% maturity for blue shark is around 4 years and 210 cm TL for males and 5 years and 220 cm TL for females (Cailliet & Goldman, 2004; Dulvy et al., 2008; Yokoi et al., 2017). Taking the upper value of the estimated survival calculated in this study (0.875), 58.6% of the male population, and 51.3% of the female population would reach the age at which 50% are mature. The same analysis was conducted for shortfin mako (upper estimated survival rate of 0.807). For this species, age and size at 50% maturity is 7.5 year and 200 cm for males and 18 years and 280 cm for females (Dulvy et al., 2008; Natanson et al., 2006; Semba et al., 2009; Yokoi et al., 2017). This information suggests that only 20.0% of the male population and 2.1% of the female population would reach the age at which 50% are mature in the North Atlantic (34% of males and 9% of the female population according to Wood et al., 2007). Given the late maturity and low fecundity of shortfin mako, these conservative results highlight the strong vulnerability of this species to industrial fisheries.

Our study focused on estimating the survival and dispersal rate of juvenile individuals of blue and shortfin mako sharks in open sea areas and fishing grounds. Results suggest that more than one half of juvenile mortality in blue shark and shortfin mako in the North Atlantic is due to fishing. By combining this information with our estimates of annual survival rates, fishing mortality (*F*) is estimated, following (*F* = (1 − *S*)*f), at 0.220 for shortfin mako and 0.095 for blue shark. As a proportion of their population size, more shortfin makos die from fishing than blue sharks. This agrees with previous studies suggesting higher fishing mortality for shortfin mako compared to blue shark. For instance, previous *F* estimated for shortfin mako ranged between 0.015–0.024 for 2012 and 0.247 for 2017 calculated by stock assessment models (ICCAT, 2012, 2017), 0.10 based on mark–recapture methods (Wood et al., 2007), and 0.19–0.56 based on satellite tagging data (Byrne et al., 2017). However, other studies predicted higher survival in the first 60 days for shortfin mako (0.884, CI 0.74–0.952; Francis et al., 2023). In the case of blue shark, *F* in the western North Atlantic Ocean ranged between 0.1 and 0.2 for the years 1965–2004, based on mark–recapture methods (Aires‐Da‐Silva et al., 2009). Although blue shark and shortfin mako have been historically captured as bycatch in Atlantic swordfish *Xiphias gladius* fisheries, during the past two decades they have also become target of industrial pelagic longliners (Camhi & Pikitch, 2008; Queiroz et al., 2016). In fact, the estimated global fishing capture of blue sharks reached 100,000 tons landed in the period 2016–2022, with a peak in 2016 (more than 110,000 t) and slight decrease over last years (FAO, 2023). The high proportion of mortality due to fishing in both blue shark and shortfin mako is not surprising given the high overlap between these species' spatial distribution and preferred fishing areas of vessels, having on average 62% and 76% of their space use, respectively, overlapped by longlines each month (Queiroz et al., 2016, 2019). Furthermore, in the North Atlantic fishing‐induced mortality (catch per unit effort) of pelagic sharks has been demonstrated to be higher where the overlap between shark space‐use hotspots and longline fishing effort is greater (Queiroz et al., 2021), which underlies the long‐term declines in abundance of these species attributed to overfishing (Pacoureau et al., 2021).

Our results suggest that more than one‐third of the tagged sharks may have moved out of the study area permanently. The long‐distance, wide‐ranging movements observed in this study and the known highly migratory nature of these sharks suggest, in agreement with previous studies, that there is a single well‐mixed population in the entire North Atlantic for both species (Schrey & Heist, 2003; Veríssimo et al., 2017), including global panmixia (Corrigan et al., 2018). Habitat selection and use of space studies of blue sharks have provided evidence for the existence of a central North Atlantic nursery where blue shark juveniles can reside for up to at least 2 years (Vandeperre et al., 2016). After birth, juveniles spatially segregate with different ontogenic movements, where females travel toward tropical latitudes and males display diverse behavioral strategies (Vandeperre et al., 2014). In the case of shortfin mako, newborns and juveniles may be dispersed over a broad geographical area from the Gulf Stream in the west (Kohler et al., 2002) to the African coast in the east (Dinkel & Sánchez‐Lizaso, 2020). In this work, differences between sexes or sizes were not explored due to data limitations, although they represent a natural next step. Our results show that two thirds stayed in the study area, indicating that there are preferred areas of space‐use hotspots in the North Atlantic, explaining and support the findings about overlap between fishing effort and blue and shortfin mako space use (Queiroz et al., 2016, 2019).

The tag‐reporting rate (percentage of returning information when a tagged shark is caught) in our study was relatively high (0.794), considering the possible loss of information during long‐distance movements and lack of motivation for reporting by some fishers, and was similar to reporting rates of coastal shark species like the sand tiger shark (*Carcharias taurus*; 0.753; Dicken et al., 2006). Given the highly migratory nature of the blue shark and shortfin mako a lower rate could be expected; however, the result is consistent with high spatial overlap between fishing activity of longliners (between 67% and 76% per overlap per month) and the range of oceanic shark species where higher tag‐reporting rates are feasible (Mucientes et al., 2022; Pacoureau et al., 2021; Queiroz et al., 2016, 2019). The recovery rate for both species was also relatively high (18.11% and 8.84% for makos and blue sharks) compared with other studies in Atlantic Ocean that reported recovery rates ranging from 9.4% and 13.5% for mako, and from 4.9% and 11.9% for blue shark (Casey & Kohler, 1992; Kohler & Turner, 2001, 2019; Wood et al., 2007). However, we cannot disregard the fact that some tags may have shed from the sharks. Previous estimates indicate a low tag‐shedding rate of 0.11 per year for blue shark (Aires‐Da‐Silva et al., 2009) and 0.259 for shortfin mako due to corrosion, constant drag, and poorly applied tags (Wood et al., 2007). In our modeling approach, we included several unobservable states, so tag loss events could be reasonably considered as transitions toward such states, but they were also confounded with other states, for instance either permanent emigration or unobserved death. A high rate of tag loss might have increased our estimates of emigration out of the study area and hence mortality also.

In comparison with global datasets available (Kohler & Turner, 2019), the relatively small and sparse dataset of tag‐recovery data of this study, that is, unique in being focused on juveniles in heavily fished areas, did not allow us to develop more complex models that included a sex effect or interactions between state and time. Instead, we focused on obtaining single (mean) values of our biological parameters of interest, and so we kept model structure as simple as possible to obtain as reliable a set of estimates as was possible with the data limitations. The results provided here are therefore a starting point for further studies as additional data are collected in the future. Nevertheless, our estimates provide reference demographic estimate values relevant to quantitative analyses of juveniles aimed at providing valuable information to conserve and manage stocks of threatened elasmobranch species, particularly oceanic species that have declined over the last few decades due to overfishing.

Conservation efforts in the North Atlantic have focused on banning landings, an obligation to release individual shortfin makos that are brought alongside the vessel alive, and TAC for blue shark. Yet, both species are still considered threatened by IUCN (Rigby, Barreto, Carlson, Fernando, Fordham, Francis, Herman, et al., 2019; Rigby, Barreto, Carlson, Fernando, Fordham, Francis, Jabado, et al., 2019). The results of our study (high fishing mortality rates, particularly among juveniles, and low chances to reach maturity) support the need to maintain existing conservation measures and continue monitoring the catches of blue shark and shortfin mako. Furthermore, in addition to maintaining existing measures, decision‐makers and managers should propose and implement strategies aimed at reducing the spatial overlap of threatened sharks and fishing. This includes implementing efficient bycatch mitigation measures and static/dynamic time–area closures in the open ocean to reduce the interaction of fisheries with juvenile blue and shortfin mako sharks.

## AUTHOR CONTRIBUTIONS

**Gonzalo Mucientes:** Conceptualization (lead); data curation (lead); formal analysis (supporting); funding acquisition (supporting); investigation (equal); methodology (equal); resources (equal); software (supporting); validation (equal); visualization (equal); writing – original draft (lead); writing – review and editing (equal). **Albert Fernández‐Chacón:** Conceptualization (equal); data curation (equal); formal analysis (lead); investigation (equal); software (lead); visualization (equal); writing – original draft (equal). **Nuno Queiroz:** Funding acquisition (equal); methodology (equal); resources (equal); supervision (equal); writing – review and editing (supporting). **David W. Sims:** Funding acquisition (equal); methodology (equal); resources (equal); supervision (equal); writing – review and editing (equal). **David Villegas‐Ríos:** Formal analysis (supporting); investigation (equal); methodology (equal); supervision (equal); writing – review and editing (equal).

## Supporting information


Appendix S1

## Data Availability

The data (shark recoveries) that supports the findings of this study are available in the Tables 2 and 3 of this article. All tagging information (tagged sharks) of this study are available from the corresponding author upon reasonable request.
