# Supplementary material for: Juvenile survival and movements of two threatened oceanic sharks in the North Atlantic Ocean inferred from tag‐recovery data
Source: Ecol Evol. 2023 Jun 21;13(6):e10198. doi: 10.1002/ece3.10198 (PMC10283031; doi:10.1002/ece3.10198)
Supplement: Supplementary file 1 — Appendix S1 [file ECE3-13-e10198-s001.docx]

**Appendix A**

**Specification of the multi-event modelling approach in program E-SURGE**

Multi-event models were built in several stages using program E-SURGE (Choquet and Nogue 2010). Each step represents one different biological/observational process (“D”, “S”, “M” and “E”, see below). This is done by means of row-stochastic matrices, i.e. each row corresponds to a multinomial. Consequently, the total of cell probabilities is 1. Because of this constraint, one and only one cell probability in each row will be calculated as the complement to 1 of the others. This particular cell is denoted with a ‘*’ symbol. Inactive cells, i.e. cells whose associated probability is structurally 0 are denoted with a ‘-’ symbol. An active cell receives an arbitrary letter. Note that the same letter/symbol in two cells does not mean that the two values should be equal.

The individual states considered are:

I: alive in the study area

O: alive outside the study area

DFI: newly dead by fishing in the study area

DUI: newly dead by other (unknown) causes in the study area

DUO: newly dead outside the study area

†: dead for long time

The possible events (field observations) are:

0: not encountered

1: captured for the first time

2: recovered dead

The steps included in the model pattern (transition matrices) are:

I: Initial state*

D: Dispersal (movement transitions)

S: Survival

M: Causes of mortality

E: Detection (event probabilities)

* This step is required to initiate the sequence of state transitions in program E-SURGE.

The symbols for parameters are:

**y**, Initial state probabilities

***d***, Dispersal probabilities

***s***, Survival probabilities

***m***, fishing mortality proportions

***p***, recapture probability

***r***, reporting rate

Model pattern

I: Initial State probabilities (newly dead states “DFI”, “DUI”, “DUO” are empty and state “†” is not represented; this is because dead states cannot be initial states in the individual encounter history)

| I | O | DFI | DUI | DUO |
| --- | --- | --- | --- | --- |
| **y** | ***** | **-** | **-** | **-** |

D: The following matrix describes the dispersal process. Note that dispersal transitions occur only when the animal is alive, so only cells corresponding to alive states will be active. At this step, transitions between dead states have no explicit parameters, only the “*“ symbol denoting that if a shark is in a dead state, it will remain as such during this step (i.e. dispersal is not possible for dead individuals).

| From/to | I | O | DFI | DUI | DUO | † |
| --- | --- | --- | --- | --- | --- | --- |
| I | * | *d* | - | - | - | - |
| O | *d* | * | - | - | - | - |
| DFI | - | - | * | *-* | - | - |
| DUI | - | - | *-* | * | - | - |
| DUO | - | - | - | - | * | - |
| † | - | - | - | - | - | * |

S: this step allows calculation of survival probabilities, which are conditional on dispersal (see also main text). In this step, the complementary cell denoted with symbol “*” represents total mortality (1-*s*); in our case, total mortality associated to each living state is estimated by transitions to two complementary “newly dead” states: “DI” and “DO” (see below). At this stage, the newly dead state corresponding to individuals dying in the study area (“DI”), is not split in mortality causes (but see next step). Survival is not possible for individuals that were already in dead states DFI, DUI and DUO, so in this step, they will move to the “long time dead” state (†), as indicated by the “*” symbol in the corresponding cells (last column).

| From/to | I | O | DI | DO | † |
| --- | --- | --- | --- | --- | --- |
| I | *s* | *-* | *** | *-* | - |
| O | *-* | *s* | *-* | *** | - |
| DFI | *-* | *-* | *-* | *-* | * |
| DUI | *-* | *-* | *-* | *-* | * |
| DUO | *-* | *-* | *-* | *-* | * |
| † | - | - | - | - | * |

M: Cause-specific mortality proportions can be estimated at this step. These transitions are conditional on the individual having died (i.e. 1-*s*) in the previous step and are directed to the different causes of mortality (Fishing or unknown). By definition, this step does not apply to living individuals, so the cells corresponding to the alive states contain the symbol “*” indicating that those that have survived in the previous step remain alive. Transitions to states “DUI” and “DUO”, which reflect unknown cause of death, are not represented by parameter *m* but calculated as the complement (1-*m*) using also symbol “*”. For those sharks dying in the study area (where recoveries were obtained), *m* estimates the proportion of deaths due to fishing (i.e. transition from “DI” to “DFI” state), whereas its complement (1-*m*) estimates the proportion of deaths due to other sources of mortality, excluding fishing.

| From/to | I | O | DFI | DUI | DUO | † |
| --- | --- | --- | --- | --- | --- | --- |
| I | * | - | - | - | - | - |
| O | - | * | - | - | - | - |
| DI | - | - | *m* | * | - | - |
| DO | - | - | - | - | * | - |
| † | - | - | - | - | - | * |

E: This is the matrix for event probabilities. Here, we distinguished individuals captured alive from those recovered dead and represented each encounter probability accordingly, using *p* for live captures and *r* for the probability of reporting dead fish. Despite none of the reencountered sharks were alive, *p* has to be included in the matrix, as all sampled individuals were tagged and released alive when first captured; *p* is then fixed to zero after 1^st^ capture using the “Initial Values-Fixed Values” step in E-SURGE. The complement of detection is non-detection, so cells corresponding to event “0” are filled with symbol “*”. Because states “DUI”, “DUO”, and “†” are always unobservable, only event “0” column is filled in their corresponding rows.

|  |  |  |  |
| --- | --- | --- | --- |
| From/to | 0 | 1 | 2 |
| I | * | *p* | *-* |
| O | * | *-* | *-* |
| DFI | * | *-* | *r* |
| DUI | * | *-* | *-* |
| DUO | * | *-* | *-* |
| † | * | - | - |

Event codes:

0: not encountered

1: captured for the first time

2: recovered dead
